# A 6 month extension trial evaluating safety and efficacy of ferric derisomaltose in patients with iron deficiency anemia: The FERWON‐EXT trial

**DOI:** 10.1002/ajh.25920

**Published:** 2020-07-31

**Authors:** Maureen M. Achebe, John Glaspy, Philip A. Kalra, Michael Auerbach, Lars L. Thomsen, Sunil Bhandari

**Affiliations:** ^1^ Divison of Hematology, Brigham and Women's Hospital Dana Farber Cancer Institute, Harvard Medical School Boston Massachusetts USA; ^2^ Department of Medicine, Division of Hematology Oncology UCLA School of Medicine Los Angeles California USA; ^3^ Department of Renal Medicine Salford Royal NHS Foundation Trust Salford UK; ^4^ Department of Medicine Georgetown University School of Medicine Washington District of Columbia USA; ^5^ Department of Clinical and Non‐Clinical Research Pharmacosmos A/S Holbaek Denmark; ^6^ Department of Renal Medicine Hull University Teaching Hospitals NHS Trust Kingston upon Hull UK

To the Editor:

Iron deficiency anemia (IDA) is a common health problem affecting more than 2 billion people worldwide. The ability to administer high dose intravenous (IV) iron facilitates the management in clinical conditions where demands for iron are high, as well as in which oral iron is ineffective, not tolerated, or harmful.

Intravenous iron has been associated with concerns of potential hypersensitivity reactions,^1^ and although such reactions are rare, there is caution with use due to perceived risk of potential reactions. Randomized clinical trials (RCT) evaluating and comparing the safety of IV iron with a co‐primary endpoint of the incidence of serious or severe hypersensitivity reactions have been performed in the FERWON‐IDA^2^ and FERWON‐NEPHRO^3^ trials. They compared ferric derisomaltose (FDI), also known as iron isomaltoside 1000, with iron sucrose (IS). The two trials included 3050 patients with IDA with either different clinical diagnoses (FERWON‐IDA trial)^2^ or non‐dialysis‐dependent CKD (FERWON‐NEPHRO trial).^3^ The co‐primary endpoints were achieved in both trials with a low frequency of serious or severe hypersensitivity reactions of 0.3% with FDI, a more pronounced increase in hemoglobin (Hb) with FDI during the first weeks, and confirmation of non‐inferiority at week eight when compared to IS.^2^, ^3^ Similarly, in the PROVIDE trial, which compared FDI and IS in 511 patients with IDA, FDI was superior to IS at increasing patients' Hb (proportion of patients with Hb increase 2 g/dL), and both treatments were well tolerated with only 0.6% experiencing serious adverse reactions.^4^

Most trials with FDI and other IV iron formulations have been 4‐12 weeks in duration, and safety trials with a longer duration are warranted to investigate long‐term safety after re‐dosing. Herein we present a 6 month extension trial (FERWON‐EXT) enrolling patients from three randomized, comparative, open‐label trials with FDI performed in the USA ‐ the PROVIDE,^4^ FERWON‐IDA,^2^ and FERWON‐NEPHRO^3^ trials. The aim of the FERWON‐EXT trial was to evaluate the safety and efficacy of FDI re‐dosing.

The primary endpoint was the number of adverse drug reactions (ADRs). The secondary safety endpoints included incidence of adjudicated serious or severe hypersensitivity reactions, composite cardiovascular adverse events (AEs), hypophosphatemia (s‐phosphate 2.0 mg/dL), and change in Hb, s‐ferritin, and transferrin saturation from baseline to week two, and months three and six. Adjudication of serious or severe hypersensitivity reactions and composite cardiovascular AEs was performed by an independent Clinical Endpoint Adjudication Committee. The hypersensitivity terms were defined by a standardized set of Medical Dictionary for Regulatory Activities (MedDRA) terms.^2^, ^3^

A group of 193 patients from the three previous RCTs were screened, of whom 103 were enrolled and 94 (91%) completed the trial. A total of 101 patients received one dose of 1000 mg FDI. One patient experienced a transient episode of back pain during the infusion and received only 350 mg.

A total of seven ADRs in 5/102 (4.9%) patients were reported. No ADR term was reported more than once. The proportion of patients with ADRs (Clopper‐Pearson 95% CI) was 0.05 (0.02; 0.11). No serious ADRs, or serious or severe hypersensitivity reactions were reported.

Six events in 6/102 (5.9%) were confirmed as cardiovascular events, primarily in patients with CKD (four events in four patients). None of the adjudicated and confirmed cardiovascular events were assessed as related to FDI.

Hypophosphatemia was reported in 8/102 (7.8%) and none of these patients had CKD. Of these events, one was reported as a mild, non‐serious ADR, whereas the others were not reported as clinically significant. None developed severe hypophosphatemia (*s*‐phosphate 1.0 mg/dL).

The mean (±SD) Hb level increased significantly from baseline (9.60 ± 1.52 g/dL) to week two (10.88 ± 1.29 g/dL), month three (11.38 ± 1.29 g/dL), and month six (11.06 ± 1.60 g/dL) with a peak at month three (Figure 1). The mean (±SD) *s*‐ferritin level increased significantly from baseline (68.2 ± 87.2 ng/mL) to week two (323 ± 227 ng/mL), month three (153 ± 188 ng/mL), and month six (157 ± 218 ng/mL) with a peak at week two (Figure 1). The mean (±SD) transferrin saturation level increased significantly from baseline (14.5 ± 13.2%) to week two (22.6 ± 8.8%) and month three (19.0 ± 10.3%), whereas there was no statistical difference at month six (16.5 ± 9.3%) (Figure 1).

**FIGURE 1:**
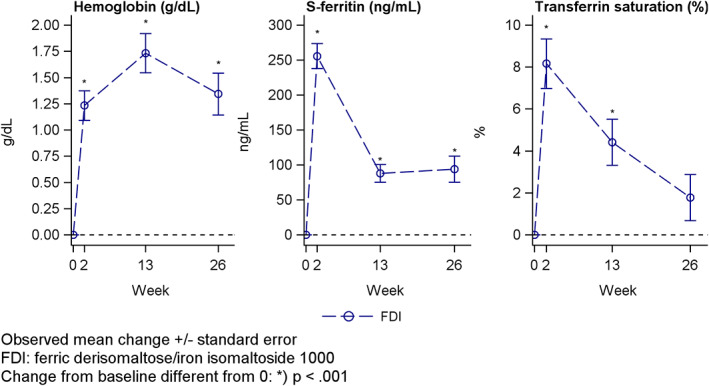
Change in hemoglobin (g/dL), *s*‐ferritin (ng/mL) and transferrin saturation (%) from baseline to week two and month three and six (intention to treat analysis set)

In this extension trial, consisting of patients from the PROVIDE,^4^ FERWON‐IDA,^2^ and FERWON‐NEPHRO^3^ trials, the incidence of ADRs was low and similar to what was reported in the FERWON‐NEPHRO^3^ trial (4.7%) and lower than in the PROVIDE^4^ (22.5%) and FERWON‐IDA^2^ (12.5%) trials. Of the 102 participants, seven (7%) had experienced an ADR in the previous “lead‐in” RCT. Furthermore, the reported ADRs were mild or moderate in severity, and no serious ADRs or serious or severe hypersensitivity reactions were reported. Re‐dosing with FDI did not adversely affect the risk of developing serious or severe reactions. A low incidence of blindly adjudicated and confirmed serious or severe hypersensitivity reactions of 0.3% was observed with FDI treatment in both the FERWON‐IDA^2^ and FERWON‐NEPHRO^3^ trials. These data are supported by a recent published analysis of the two head‐to‐head PHOSPHARE trials comparing hypersensitivity reactions in patients with IDA treated with FDI or ferric carboxymaltose (FCM).^5^ In the PHOSPHARE trials the incidence of serious or severe hypersensitivity reactions was a pre‐defined secondary endpoint and the results showed an incidence of 0.8% for FDI and 1.7% for FCM.^5^

The most extensive and robust approach conducted to date to assess the risk of serious or severe hypersensitivity reactions with FDI, FCM, and IS included a total of 8599 patients from 21 RCTs.^6^ There was a low incidence of serious or severe hypersensitivity reaction with all IV iron products. Different statistical methods were used with the primary analysis (Bayesian inference) showing a mean odds ratio of 0.41 for FDI vs FCM, indicating a 59% lower risk of experiencing a serious or severe hypersensitivity reaction with FDI relative to FCM.^6^ The mean odds ratio was 0.51 for FDI vs IS, indicating a 49% lower risk of experiencing a serious or serious hypersensitivity reaction with FDI relative to IS.^6^

The incidence of adjudicated and confirmed composite cardiovascular AEs in the present trial was 5.9%. Most of the events occurred in patients from the FERWON‐NEPHRO trial who were older, and had a higher incidence of cardiac disorders as compared to patients in the other two lead‐in trials, emphasizing that patients with CKD have a higher risk of cardiovascular events compared to a more broad IDA population.

The incidence of hypophosphatemia in this trial was 7.8% which was similar to the frequency in the PHOSPHARE trials, in which patients treated with FDI had a significantly lower incidence of hypophosphatemia than those treated with FCM (8.0% vs 74.4%, *P* < .001).^5^ In the PHOSPHARE trials, FCM induced high rates of intact fibroblast growth factor 23 which resulted in hypophosphatemia through renal phosphate wasting. The hypophosphatemia induced by FCM was frequently severe with s‐phosphate 1.0 mg/dL in 11.3% of the FCM group vs none in the FDI group (*P* < .001), and hypophosphatemia often persisted at the end of follow up at day 35 (43.0% in the FCM group vs 0.9% in the FDI group).^5^ The current trial showed that re‐dosing with FDI did not increase the incidence of hypophosphatemia.

While the primary objective and endpoints of FERWON‐EXT were safety, hematopoietic responses were also assessed. Mean Hb, *s*‐ferritin, and transferrin saturation increased significantly from baseline and peaked 2 weeks (*s*‐ferritin, and transferrin saturation) or 3 months (Hb) after dosing. Note, FDI is the only IV iron with an approval in the US for dosing of 1000 mg IV iron in one infusion, and administration of single doses above 1000 mg for patients with a bodyweight >50 kg (20 mg/kg) in the EU. The efficacy of FDI is well documented in RCTs, and FDI is at least as effective as FCM in correcting IDA^6^ and provides a faster and more pronounced hematological effect than IS.^2^, ^3^, ^4^

The strengths of the trial were inclusion of a broad population across a wide range of IDA etiologies, including pre‐menopausal women with menorrhagia who were otherwise healthy, and CKD patients of whom 50% were 65 years or older. Iron deficiency anemia was confirmed in all patients based upon Hb and *s‐*ferritin, transferrin saturation or both, and those previously experiencing ADRs with FDI treatment were not excluded. Finally, the trial duration was 6 months, allowing for assessment of longer‐term safety.

In conclusion, these data demonstrated that re‐dosing with a single IV dose of 1000 mg FDI was well tolerated and provided a fast improvement in Hb which was sustained 6 months after dosing. All dosed patients, except one, received the full FDI infusion without interruption. Adverse drug reactionss were reported in 4.9% of the patients, and all were mild or moderate in severity and none were serious. No serious or severe hypersensitivity reaction occurred.

## CONFLICT OF INTEREST

Maureen M. Achebe has been on scientific advisory boards for Pharmacosmos A/S, AMAG, Global blood therapeutics and Fulcrum pharmaceuticals.

John Glaspy has been an advisor to AMAG Pharmaceuticals.

Philip A. Kalra has received personal fees and non‐financial support from Pharmacosmos A/S, grants and personal fees from Vifor Pharma, and grants from Astellas.

Michael Auerbach receives research funding for data management from AMAG Pharmaceuticals.

Lars L. Thomsen is employed by Pharmacosmos A/S.

Sunil Bhandari has received honorarium, consultancy fees, membership advisory board, and travel funding from Pharmacosmos A/S, Vifor Pharma, and Astellas.

This work was funded by Pharmacosmos A/S and the investigators/institutions received a fee per patient.
